# Editorial: Nutrient Interactions in Plants

**DOI:** 10.3389/fpls.2021.782505

**Published:** 2021-11-23

**Authors:** Francisco Javier Romera, Ping Lan, Jorge Rodríguez-Celma, Rafael Pérez-Vicente

**Affiliations:** ^1^Department of Agronomy-Universidad de Córdoba (DAUCO-María de Maeztu Unit of Excellence), Edificio Celestino Mutis (C-4), Campus de Excelencia Internacional Agroalimentario de Rabanales CeiA3, Universidad de Córdoba, Córdoba, Spain; ^2^State Key Laboratory of Soil and Sustainable Agriculture, Institute of Soil Science, Chinese Academy of Sciences, Nanjing, China; ^3^Plant Nutrition Department, Aula Dei Experimental Station, CSIC (Consejo Superior de Investigaciones Científicas), Zaragoza, Spain; ^4^Department of Botany, Ecology and Plant Physiology, Edificio Celestino Mutis (C-4), Campus de Excelencia Internacional Agroalimentario de Rabanales CeiA3, Universidad de Córdoba, Córdoba, Spain

**Keywords:** crosstalk, mineral nutrition, nutrient, nutrient acquisition, nutrient deficiency responses, nutrient interactions

Plants, like other living organisms, require an assemblage of essential elements to synthesize their constituent compounds and for essential metabolic reactions. Besides carbon (C), hydrogen (H) and oxygen (O), plants require 14 essential mineral elements such as nitrogen (N), phosphorus (P), potassium (K), sulfur (S), magnesium (Mg), calcium (Ca), zinc (Zn), iron (Fe), copper (Cu), manganese (Mn), molybdenum (Mo), nickel (Ni), chlorine (Cl) and boron (B) (Marschner, 2012). Additionally, there are other mineral elements that are not essential for all plant species but that can be beneficial for some groups of plants, like sodium (Na; Maathuis, 2014) or silicon (Si; Tripathi et al., 2020). All these elements interact in a direct and/or indirect manner. In some cases, the deficiency or excess of one element can affect the uptake of other(s), thus conditioning their proper uptake and efficient utilization (Astolfi et al., 2021; Bernal and Kramer, 2021; Pavlovic et al., 2021; Yu et al.; Zhou et al.). For instance, S deficiency can limit Fe acquisition (Astolfi et al., 2021) while P deficiency can promote it (Figure 1; García et al.). On the other hand, P excess can diminish Zn acquisition (Yu et al.). In other cases, a scarce element, i.e., K, can be substituted by another element of similar characteristics, like Na (Mateus et al.).

**Figure 1:**
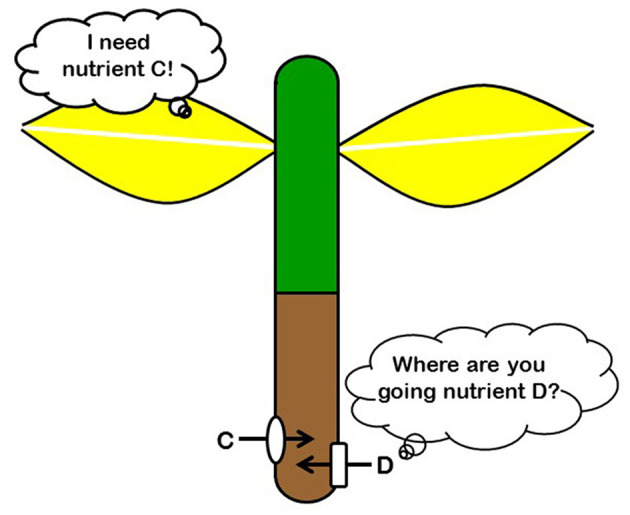
The responses to the deficiency of a particular nutrient can promote the acquisition of such a nutrient but also the acquisition of other nutrients.

This Research Topic updates recent results showing the interactions between different essential mineral nutrients, and also between essential and non-essential ones. It includes 5 reviews, 1 minireview, 1 perspective and 13 original research articles. Regarding the reviews; one is related to interactions between two essential elements, S and Fe (Astolfi et al., 2021); two are related to interactions between macro- and micro-nutrients (Fan et al., 2021; Kumar et al., 2021); and the other two are dedicated to interactions between beneficial elements, Si and Se, and essential ones (Pavlovic et al., 2021; Zhou et al.). The minireview deals with the interaction between N and P in the development of root nodules and cluster roots (Pueyo et al., 2021). The perspective article is devoted to describing new approaches based on computational analysis to predict interactions between proteins related to different elements (Di Silvestre et al.). Finally, within the 13 original research articles; eight of them are about the interactions between two or three elements, including non-essential ones, such as Fe-Cu, Si-Fe, Fe-P-S, S-N, Fe-Zn, K-Na, or P-Zn (Bernal and Kramer, 2021; Jian et al., 2021; Kakei et al., 2021; Li et al., 2021; Chaiwong et al.; García et al.; Suman et al.; Yu et al.); four of them are about the interactions among many nutrients, such as ionome-macronutrients, ionome-micronutrients, ionome-N (Courbet et al.; D'Oria et al.; Zhang C. et al.; Zhang J. et al.); and one is devoted to the substitution of K by Na (Mateus et al.).

The articles included in this Research Topic reflect indirect interactions between nutrients, such as those simultaneously analyzing many nutrients (Courbet et al.; D'Oria et al.; Zhang C. et al.; Zhang J. et al.), and also direct interactions, like those studying the interplay between 2 and 3 elements (Bernal and Kramer, 2021; Jian et al., 2021; Kakei et al., 2021; Li et al., 2021; Chaiwong et al.; García et al.; Mateus et al.; Suman et al.; Yu et al.). The depicted interactions occur at different steps of nutrient acquisition and translocation inside the plant. For instance, P deficiency, through organic acid release and rhizosphere acidification, can promote the mobilization of other nutrients, like Fe or Zn (Pueyo et al., 2021). In the same way, Si application can promote N and P acquisition by upregulating nitrate and phosphate transporters (Pavlovic et al., 2021). New interactions are described in this Research Topic, like the uptake of vanadium mediated by sulfate transporters whose expression was stimulated during S deprivation (Courbet et al.). In relation to the translocation of some elements, like Fe, Cu and Mn, S deficiency can negatively affect it by limiting the biosynthesis of nicotianamine, a chelating agent linked to this process (Astolfi et al., 2021).

In this Research Topic, mechanisms underlying the observed interactions are proposed. Two elements can interact because they share similar chemical properties, like K and Na (Mateus et al.). One element can participate in compounds or proteins involved in key processes related to others [i.e., S-containing metabolites participate in the synthesis of ethylene and phytosiderophores, which are in turn implicated in Fe uptake (Astolfi et al., 2021); a multicopper oxidase participates in Fe translocation (Bernal and Kramer, 2021)]. The participation of different elements in the same compounds (i.e., N and S in methionine and cysteine; Fe and S in Fe-S clusters) can also cause their interactions (Astolfi et al., 2021). Finally, the participation of the same phytohormones, signaling molecules (nitric oxide, miRNAs, peptides and others), and transcription factors in the homeostasis of different elements can explain the interactions between them (Astolfi et al., 2021; Bernal and Kramer, 2021; García et al., 2021; Kumar et al., 2021; Pueyo et al., 2021; Chaiwong et al.; García et al.; Mateus et al.). For instance, ethylene and nitric oxide upregulate both P- and Fe-acquisition genes in such a way that the deficiency of either of them, that stimulate the production of ethylene and nitric oxide, promote the acquisition of the other one (García et al.).

Interactions between nutrients can have many different consequences, depending on them being essential or beneficial, and on other factors. In this sense, it is important to point out that the interactions between nutrients greatly depend on the severity of the nutrient deficiency or excess (Astolfi et al., 2021). For instance, Si upregulates nitrate and phosphate transporters when plants are grown under limiting N and P conditions but downregulates them when grown under sufficient N and P conditions (Pavlovic et al., 2021). Besides nutrition, interactions between nutrients can affect other processes, like the accumulation of secondary metabolites (Jian et al., 2021). It is important to note the interest of the interactions between essential and non-essential elements, since non-essential elements, like Na, can partially substitute for essential ones, like K (Mateus et al.). Additionally, non-essential elements, like Si, can affect the homeostasis of essential elements (Pavlovic et al., 2021), and vice versa (Chaiwong et al.; Zhou et al.). A better knowledge of such interactions could aid in the improvement of some nutritional disorders and/or in the biofortification of some essential elements for humans and animals, like Se (Zhou et al.).

In conclusion, the better understanding of the interactions between elements (essential and non-essential) could lead to more rational fertilization practices, preventing interactions that could contribute to an unbalanced mineral nutrition of plants. This knowledge is also necessary to obtain more efficient genotypes in the acquisition of the different nutrients.

## Author Contributions

All authors listed have made a substantial, direct, and intellectual contribution to the work and approved it for publication.

## Funding

PL was funded by the National Natural Science Foundation of China (32070279). FJR and RPV were funded by the Spanish Ministry of Science and Innovation (RTI2018-097935-B-I00), the Spanish State Research Agency, through the Severo Ochoa and María de Maeztu Program for Centers and Units of Excellence in R&D (Ref. CEX2019-000968-M), and the ‘Junta de Andalucía’ (Research Groups AGR115 and BIO159).

## Conflict of Interest

The authors declare that the research was conducted in the absence of any commercial or financial relationships that could be construed as a potential conflict of interest.

## Publisher's Note

All claims expressed in this article are solely those of the authors and do not necessarily represent those of their affiliated organizations, or those of the publisher, the editors and the reviewers. Any product that may be evaluated in this article, or claim that may be made by its manufacturer, is not guaranteed or endorsed by the publisher.
